# Silicon nanocrystal growth under irradiation of electron beam

**DOI:** 10.1038/srep16682

**Published:** 2015-11-26

**Authors:** Wei-Qi Huang, Shi-Rong Liu, Zhong-Mei Huang, Tai-Ge Dong, Gang Wang, Cao-Jian Qin

**Affiliations:** 1 Institute of Nanophotonic Physics, Guizhou University, Guiyang 550025 (China); 2 State Key Laboratory of Environmental Geochemistry Institute of Geochemistry, Chinese Academy of Science Institute of Geochemistry, Guiyang 550003 (China); 3 Surface Physics Laboratory, Department of Physics, Fudan University, Shanghai 200433 (China)

## Abstract

In our experiment, it was observed that silicon nanocrystal rapidly grows with irradiation of electron beam on amorphous silicon film prepared by pulsed laser deposition, and shape of silicon nanocrystal is usually sphere in smaller nanoscale with less exposure time under electron beam, in which the quantum dots are prepared in nanoscale near 3 nm. In the electron interaction process, it was investigated that the various crystals structures in different orientations occur in the same time and the condensed structures of silicon nanocrystal are changed with different impurity atoms in silicon film.

Silicon nanocrystal has been studied intensively over the past decade^1^,^2^,^3^,^4^,^5^. The popular methods for fabricating silicon nanocrystal are self-assembly from silicon-rich silicon oxide matrices^6^,^7^,^8^ and plasma synthesis^9^,^10^,^11^,^12^. The interesting method to fabricate silicon nanocrystal is growth under photons interaction^13^,^14^,^15^. In the first case, SiO*x* (with *x* < 2) is formed by a thin-film deposition technique such as pulsed laser deposition (PLD). Subsequent high-temperature annealing of the substoichimetric film (typically 900 ~ 1100 °C) produces a phase separation between Si and SiO_2_ with the formation of Si nanoclusters. The dimensions, crystallinity and size distribution of the nanoclusters depend on the Si excess, the temperature and the annealing time^6^,^7^.

In the article, the most interesting and simplest method discovered in our experiment for fabricating silicon nanocrystal is self-assembly growth by assistance of electron interaction, in which silicon nanocrystal rapidly grows with irradiation of electron beam on amorphous silicon film prepared by PLD, and shape of silicon nanocrystal is usually sphere when crystal size is smaller with less exposure time of electron beam. The method of electronic irradiation could be used to replace the traditional annealing methods in preparing process of silicon nanocrystal. In the process, it was investigated that the condensed structures of silicon nanocrystal are changed with different impurity atoms in silicon film, for examples oxygen or Er atoms make a stronger condensed trend than doing of nitrogen or Yb atoms in impurity, in which various localized states emission was measured.

It is very interesting that the electronic irradiation promotes the growth of nanocrystal, whose physical mechanism may be from the nanoscale characteristics of electronic de Broglie wave which produces resonance to transfer energy to crystal atoms. In natural sciences many analogous structures and properties occur on different size hierarchy, for example in the nanoscale space related to electronic de Broglie wavelength and in the sub-micrometer scale related to photonic de Broglie wavelength, in which the nanosecond or femtosecond laser is used to fabricate periodic surface structures with 100 nm spatial periods on silicon^16^,^17^, and the electron irradiation is used to prepare silicon nanocrystal.

Some silicon wafers of P-type (100) oriented substrate with 1–10 Ωcm were taken on the sample stage in the combination fabrication system with pulsed laser etching (PLE) and PLD devices. A pulsed Nd:YAG laser (wavelength: 1064 nm, pulse length: 60 ns FWHM, repetition rate: 1000) was used to etch the Purcell micro-cavity on Si sample in PLE process. In the cavity, a third harmonic of pulsed Nd:YAG laser at 355 nm was used to deposit amorphous silicon film in PLD process. Then, the amorphous silicon film was exposed under electron beam with 0.5 nA/nm^2^ for 5 ~ 30 min in Tecnai G2 F20 system in Fig. 1, in which electron beam from the field-emission electron gun, accelerated by 200 KV, has higher energy and better coherent. The right bottom inset in Fig. 1 shows TEM image of edge area of electron beam spot, which indicates the irradiation area of electron beam in silicon crystallization (top region in spot). The left top inset in Fig. 1 shows the area exposed under electron beam spot whose diameter is about 300 nm. Having been irradiated under electron beam for 15 min, silicon quantum dots (Si QDs) structures are built and embedded in SiO_x_ (with *x* < 2) or Si_y_N_x_ (with *x* < 4 and y > 3) amorphous film related to oxygen or nitrogen environment respectively, in which the density of Si QDs is about 8 × 10^18^ cm^−3^.

In Fig. 2, it was observed that the nanocrystal structures of quantum dots form in the orientation of (100), (110) or (111) with the electron irradiation for 10 ~ 20 min, in which the insets show the electronic diffraction image in selection region and the fast Fourier transform (FFT) image on the polycrystalline.

It is very interesting that gradually growing process of QDs structure was observed under electron beam with increase of exposure time. For example, the QDs structure has been observed after irradiation for 10 min (TEM image in Fig. 3(a)) on amorphous Si film prepared in nitrogen gas, and the larger bulks crystals gradually grow in various orientations with increasing the exposure time under electron beam as shown in Fig. 3(b). It is discovered that the QDs size distribution is narrower (diameter is about 3–4 nm) for electronic irradiation time of 10–15 min.

In Fig. 4(a), Si–Yb QDs structures are built in the (111) orientation after irradiation under electron beam for 15 min on the Si–Yb amorphous film prepared by PLD process, the bottom inset shows the FFT image of QDs structure and the top inset shows the simulation of Si–Yb QDs structure and its density of states in which the localized states form in band gap. Figure 4(b) shows the Si–Yb crystals with larger bulks in various orientations with longer exposure time (over 30 min) under electron beam, in which a lot of defects occur in interface of polycrystalline.

We have chosen some model in order to simulate the experiment process. The electronic behavior is investigated in the work by an abinitio non-relativistic quantum mechanical analysis. The DFT calculation were carried out by using the local density approximation (LDA) and non-local gradient-corrected exchange-correlation functional (GGA) for the self-consistent total energy calculation. It is considered that both LDA and GGA underestimate the band gap for semiconductor and insulator.

In the crystallizing process under irradiation of electron beam, it was observed that condensed speed is different to form different structures of silicon nanocrystal with different impurity atoms on silicon film, for examples oxygen or Er atoms make a stronger condensed trend than doing of nitrogen or Yb atoms in impurity. In Fig. 5, TEM images show different structures of silicon nanocrystal with different impurity gas atoms after radiation of electron beam for 30 min, such as in nitrogen gas (a), in oxygen gas (b) and in SF gas (c), in which the film structure of Si–N nanocrystal is still kept, but the film structures of Si–O and Si–S nanocrystals have been broken. It is noted that the Si–N, Si–O or Si–S bonds in the interface of polycrystalline, related to the simulation structures in the insets, produce the localized states in band gap, as shown in the insets.

As shown in Fig. 6, TEM images show different structures of silicon nanocrystal with different impurity solid atoms, such as Yb atoms (a), Ge atoms (b) and Er atoms (c), in which the film structure of Si–Yb nanocrystals is still kept, but the film structures of Si–Ge and Si–Er nanocrystals have been broken. Here, it is obvious that some atoms have a stronger condensed ability, such as Er atoms.

On the silicon nanocrystal sample prepared by using irradiation of electron beam, photoluminescence (PL) spectra were investigated in oxygen impurity atoms. Figure 7(a) shows TEM image of silicon QDs embedded in SiO_x_ prepared by using irradiation of electron beam for 15 min, whose sharper PL peak at 604 nm is observed as shown in Fig. 7(b). Here, the PL peak at 604 nm with a very narrow linewidth belongs to a kind of localized states emission originating from Si – O bonds on silicon QDs.

It is interesting that on the amorphous silicon film prepared in the PLD device with Yb and Er bars, smaller Si-Yb-Er nanocrystals begin to occur after irradiation of electron beam for 10 min. And bigger Si-Yb-Er nanocrystals with various shapes appear after radiation of electron beam for 30 min, as shown in Fig. 8(a), in which the inset shows the Fourier transform image on multi-crystals structures. Electroluminescence (EL) spectra on the sample occur in optical communication window in Fig. 8(b), which have the characteristics of localized states emission of surface bonding on Si crystals. Here, the QC effect disappears and the localized states emission from defects and impurity of bigger nanocrystals interface plays a main role, whose emission model could be described in four-level system of Fig. 8(c), in which the bumping level is in the bottom states of conduction band and relaxation level is in the localized states due to Si-Yb-Er bonding on bigger nanocrystals interface.

In conclusion, various silicon nanocrystals are fabricated by using irradiation of electron beam on Si amorphous film prepared by PLD process, in which Si QDs near 3 nm diameter could be obtained by controlling exposure time of electron beam. It is interesting that the irradiation of electron beam promotes the growth of nanosilicon. Through electron beam exposure for 15 min on amorphous Si film doped with oxygen impurity atoms by PLD process, enhanced PL emission peaks are observed in visible light. And EL emission is manipulated into the optical communication window on the bigger Si-Yb-Er nanocrystals after irradiation of electron beam for 30 min. In the process, the physical phenomena and effects are very interesting, and a new way will be developed for fabrication of silicon nanostructures, which would have good application in emission materials and LED devices.

## Methods

### Preparation of amorphous silicon film

Some silicon wafers of P-type (100) oriented substrate with 1 Ωcm were taken on the sample stage in the combination fabrication system with pulsed laser etching (PLE) and pulsed laser deposition (PLD) devices. A pulsed Nd:YAG laser (wavelength: 1064 nm, pulse length: 60 ns FWHM, repetition rate: 1000) was used to etch the Purcell micro-cavity on Si sample in PLE process. In the cavity, a third harmonic of pulsed Nd:YAG laser at 355 nm was used to deposit the amorphous silicon film in PLD process.

### Fabrication of silicon quantum dots under irradiation of electron beam

The amorphous silicon film was exposed under electron beam with 0.5 nA/nm^2^ for 5 ~ 30 min in Tecnai G2 F20 system, in which electron beam from field-emission electron gun, accelerated by 200 KV, has higher energy and better coherence. After irradiation under electron beam for 15 min, silicon quantum dots (Si QDs) structures are built and embedded in SiO_x_ (with *x* < 2) or Si_y_N_x_ (with *x* < 4 and y > 3) amorphous film related to oxygen or nitrogen gas tube respectively in the PLD device.

### Transmission electron microscope (TEM) analysis

In the TEM (JEM-2000FX) image, the silicon quantum dots and the silicon nanostructures were observed in the silicon amorphous films with impurity, and the compositions were measured on the samples by using analysis in X-ray energy spectra.

### Photoluminescence (PL) measurement

PL spectra of the samples were measured under the 514 nm excitation by using RENISHAW Micro-Raman Systems at room temperature.

### Electroluminescence (EL) measurement

EL spectra were measured on the sample whose surface was deposited the ITO film for positive pole and bottom side was deposited the Au film for negative pole.

### Annealing process

The samples were sent into the annealing furnace filled with nitrogen atmosphere to make annealing at 1050 °C for 10 min, 15 min or 20 min. The PL spectra show that annealing time of 20 min is suitable for localized states emission.

## Additional Information

**How to cite this article**: Huang, W.-Q. *et al.* Silicon nanocrystal growth under irradiation of electron beam. *Sci. Rep.* **5**, 16682; doi: 10.1038/srep16682 (2015).

## Figures and Tables

**Figure 1:**
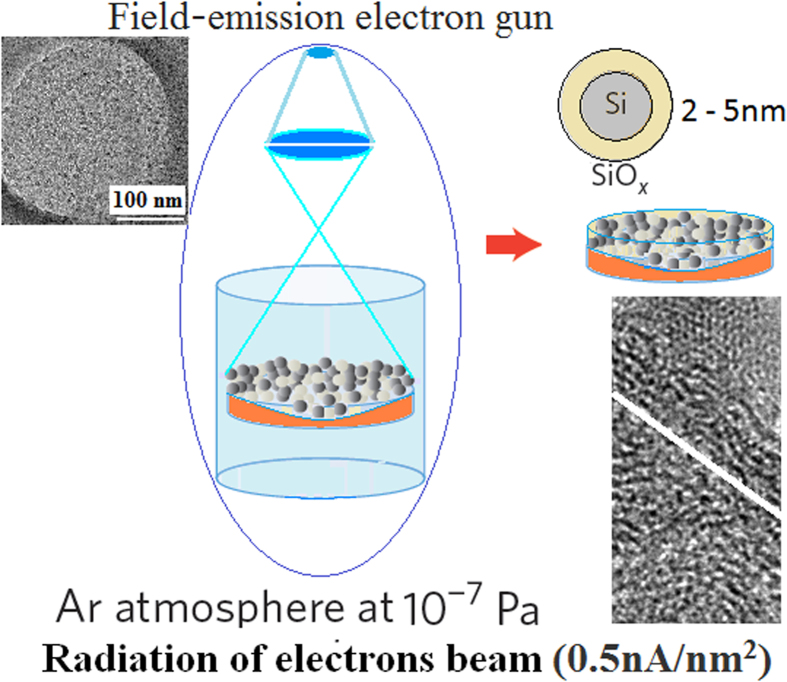
Fabrication system with electron beam irradiation device, in which the right bottom top inset shows TEM image of edge area of electron beam spot on amorphous Si film, and The left top inset shows the area exposed under electron beam spot whose diameter is about 300 nm.

**Figure 2:**
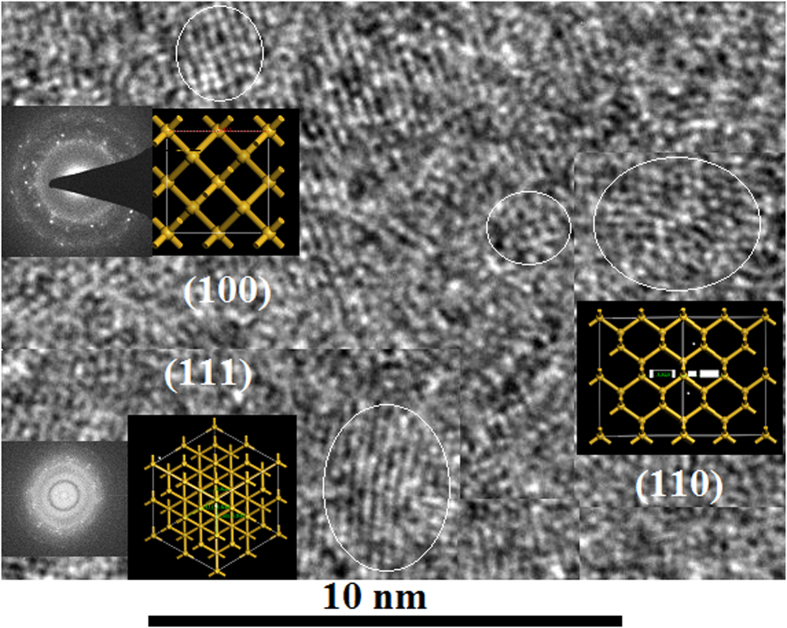
TEM images of Si QDs, in which the top insets show nanocrystal structures of QDs in various orientations, their simulation structures and their diffraction patterns (FFT images).

**Figure 3:**
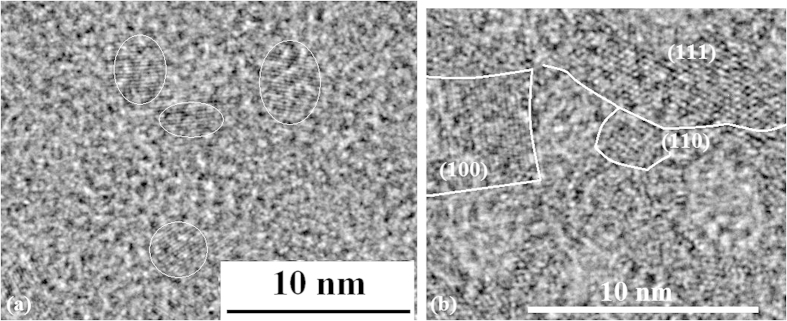
(**a**) TEM images of Si QDs prepared by irradiation of electron beam for 10 min on amorphous Si film prepared in nitrogen gas (**b**) TEM image of larger bulks Si crystals, which gradually grow in various orientations with increasing the exposure time under electron beam.

**Figure 4:**
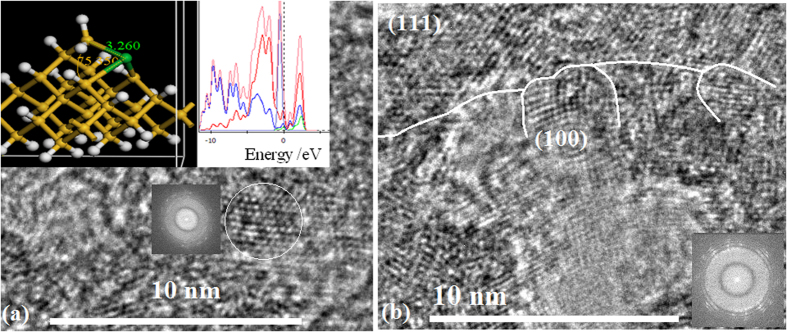
(**a**) TEM image of Si–Yb QDs structures are built in the (111) orientation after irradiation under electron beam for 15 min on the Si–Yb amorphous film prepared by PLD process, in which the bottom inset shows the FFT image of QDs structure and the top inset shows the simulation of Si–Yb QDs structure and its density of states (**b**) TEM image of Si–Yb crystals with larger bulks in various orientations with longer exposure time (over 30 min) under electron beam, in which a lot of defects occur in interface of polycrystalline.

**Figure 5:**
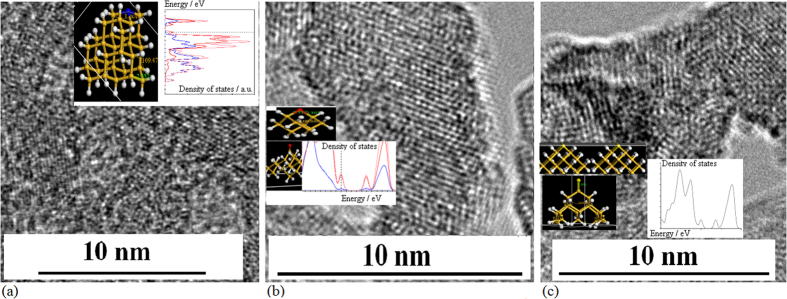
TEM images of different silicon nanocrystals prepared in different impurity gas atoms after irradiation of electron beam for 30 min, in which the insets show their simulation structures and their density of states (**a**) TEM image of Si–N nanocrystals with various shapes in amorphous film after irradiation of electron beam for 30 min, in which the film structure is still kept (**b**) TEM image of Si–O nanocrystals with various shapes in amorphous film after irradiation of electron beam for 30 min, in which the film structure has been broken because of stronger condensed ability of O atoms (**c**) TEM image of Si–S nanocrystals with various shapes in amorphous film after irradiation of electron beam for 30 min, in which the film structure has been broken because of stronger condensed ability of S atoms.

**Figure 6:**
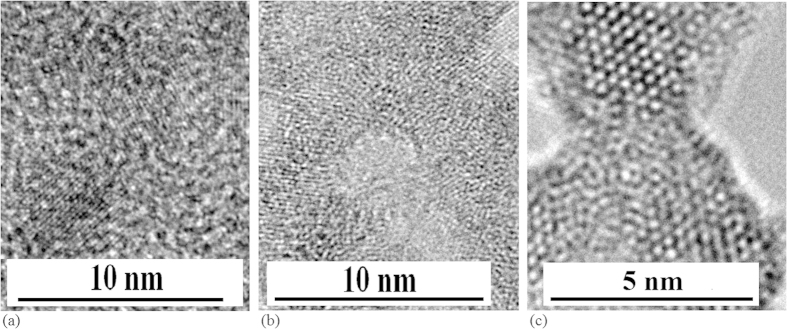
TEM images of different silicon nanocrystals prepared in different impurity solid atoms after irradiation of electron beam for 30 min (**a**) TEM image of Si–Yb nanocrystals with various shapes in amorphous film after irradiation of electron beam for 30 min, in which the film structure is still kept (**b**) TEM image of Si–Ge nanocrystals with various shapes in amorphous film after irradiation of electron beam for 30 min, in which the film structure has been broken due to stronger condensed ability of Ge atoms (**c**) TEM image of Si–Er nanocrystals with various shapes in amorphous film after irradiation of electron beam for 30 min, in which the film structure has been broken because of stronger condensed ability of Er atoms.

**Figure 7:**
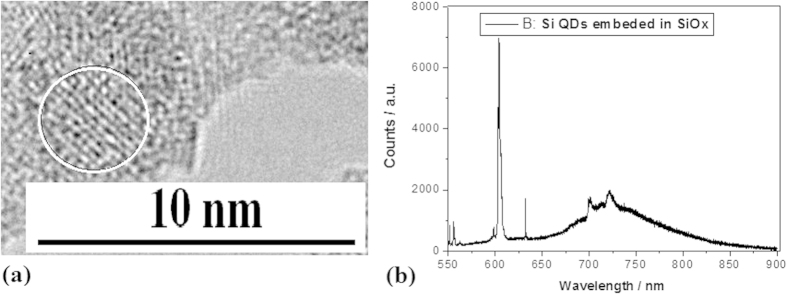
TEM image of Si QDs embedded in SiO_x_ and their PL spectra (**a**) TEM image of silicon QDs embedded in SiO_x_ prepared by using irradiation of electron beam for 15 min (**b**) PL peaks on the Si QDs oxidized sample prepared with irradiation of electron beam for 15 min.

**Figure 8:**
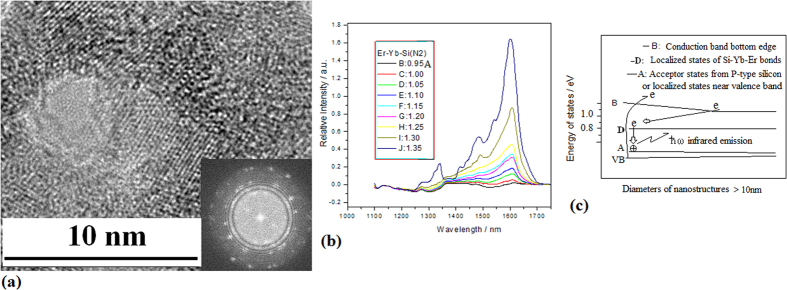
TEM image of Si -Yb-Er QDs embedded in Si_y_N_x_ and their EL spectra (**a**) TEM image of bigger Si-Yb-Er nanocrystals with various shapes embedded in Si_y_N_x_ prepared by using irradiation of electron beam for 30 min (**b**) EL emission in optical communication window on the bigger Si-Yb-Er nanocrystals sample prepared in nitrogen with irradiation of electron beam for 30 min, in which stimulated emission characteristics are observed near 1600 nm (**c**) Four-level emission model involving bumping states in conduction band bottom and localized states from Si-Yb-Er bonds on surface of bigger nanocrystals.
